# Supplementary material for: An empirical survey of data augmentation for time series classification with neural networks
Source: PLoS One. 2021 Jul 15;16(7):e0254841. doi: 10.1371/journal.pone.0254841 (PMC8282049; doi:10.1371/journal.pone.0254841)
Supplement: S1 Table — (PDF) [file pone.0254841.s001.pdf]

An empirical survey of data augmentation for time series classification with neural networks

Brian Kenji Iwana[1*], Seiichi Uchida[1]

1 Department of Advanced Information Technology, Kyushu University, Fukuoka, Japan

* iwana@ait.kyushu-u.ac.jp

S1 Table. Details of the Datasets

Name	Type	# Classes	# Time Steps	# Train	# Test
ACSF1	Device	10	1460	100	100
Adiac	Image	37	176	390	391
AllGestureWiimoteX	Sensor	10	≤500	300	700
AllGestureWiimoteY	Sensor	10	≤500	300	700
AllGestureWiimoteZ	Sensor	10	≤500	300	700
ArrowHead	Image	3	251	36	175
Beef	Spectro	5	470	30	30
BeetleFly	Image	2	512	20	20
BirdChicken	Image	2	512	20	20
BME	Simulated	3	128	30	150
Car	Sensor	4	577	60	60
CBF	Simulated	3	128	30	900
Chinatown	Traffic	2	24	20	343
ChlorineConcentration	Sensor	3	166	467	3840
CinCECGTorso	Sensor	4	1639	40	1380
Coffee	Spectro	2	286	28	28
Computers	Device	2	720	250	250
CricketX	Motion	12	300	390	390
CricketY	Motion	12	300	390	390
CricketZ	Motion	12	300	390	390
Crop	Image	24	46	7200	16800
DiatomSizeReduction	Image	4	345	16	306
DistalPhalanxOutlineAgeGroup	Image	3	80	400	139
DistalPhalanxOutlineCorrect	Image	2	80	600	276
DistalPhalanxTW	Image	6	80	400	139
DodgerLoopDay	Sensor	7	288	78	80
DodgerLoopGame	Sensor	2	288	20	138
DodgerLoopWeekend	Sensor	2	288	20	138
Earthquakes	Sensor	2	512	322	139
ECG200	ECG	2	96	100	100
ECG5000	ECG	5	140	500	4500
ECGFiveDays	ECG	2	136	23	861

Name	Type	# Classes	# Time Steps	# Train	# Test
ElectricDevices	Device	7	96	8926	7711
EOGHorizontalSignal	EOG	12	1250	362	362
EOGVerticalSignal	EOG	12	1250	362	362
EthanolLevel	Spectro	4	1751	504	500
FaceAll	Image	14	131	560	1690
FaceFour	Image	4	350	24	88
FacesUCR	Image	14	131	200	2050
FiftyWords	Image	50	270	450	455
Fish	Image	7	463	175	175
FordA	Sensor	2	500	3601	1320
FordB	Sensor	2	500	3636	810
FreezerRegularTrain	Sensor	2	301	150	2850
FreezerSmallTrain	Sensor	2	301	28	2850
Fungi	HRM	18	201	18	186
GestureMidAirD1	Trajectory	26	$\leq$360	208	130
GestureMidAirD2	Trajectory	26	$\leq$360	208	130
GestureMidAirD3	Trajectory	26	$\leq$360	208	130
GesturePebbleZ1	Sensor	6	$\leq$455	132	172
GesturePebbleZ2	Sensor	6	$\leq$455	146	158
GunPoint	Motion	2	150	50	150
GunPointAgeSpan	Motion	2	150	135	316
GunPointMaleVersusFemale	Motion	2	150	135	316
GunPointOldVersusYoung	Motion	2	150	136	315
Ham	Spectro	2	431	109	105
HandOutlines	Image	2	2709	1000	370
Haptics	Motion	5	1092	155	308
Herring	Image	2	512	64	64
HouseTwenty	Device	2	2000	40	119
InlineSkate	Motion	7	1882	100	550
InsectEPGRegularTrain	EPG	3	601	62	249
InsectEPGSmallTrain	EPG	3	601	17	249
InsectWingbeatSound	Sensor	11	256	220	1980
ItalyPowerDemand	Sensor	2	24	67	1029
LargeKitchenAppliances	Device	3	720	375	375
Lightning2	Sensor	2	637	60	61
Lightning7	Sensor	7	319	70	73
Mallat	Simulated	8	1024	55	2345
Meat	Spectro	3	448	60	60
MedicalImages	Image	10	99	381	760
MelbournePedestrian	Traffic	10	24	1194	2439
MiddlePhalanxOutlineAgeGroup	Image	3	80	400	154
MiddlePhalanxOutlineCorrect	Image	2	80	600	291
MiddlePhalanxTW	Image	6	80	399	154
MixedShapesRegularTrain	Image	5	1024	500	2425
MixedShapesSmallTrain	Image	5	1024	100	2425
MoteStrain	Sensor	2	84	20	1252
NonInvasiveFetalECGThorax1	ECG	42	750	1800	1965
NonInvasiveFetalECGThorax2	ECG	42	750	1800	1965
OliveOil	Spectro	4	570	30	30
OSULeaf	Image	6	427	200	242
PhalangesOutlinesCorrect	Image	2	80	1800	858
Phoneme	Sensor	39	1024	214	1896

**Name**	**Type**	**# Classes**	**# Time Steps**	**# Train**	**# Test**
PickupGestureWiimoteZ	Sensor	10	$\leq$361	50	50
PigAirwayPressure	Hemodynamics	52	2000	104	208
PigArtPressure	Hemodynamics	52	2000	104	208
PigCVP	Hemodynamics	52	2000	104	208
PLAID	Device	11	$\leq$1344	537	537
Plane	Sensor	7	144	105	105
PowerCons	Power	2	144	180	180
ProximalPhalanxOutlineAgeGroup	Image	3	80	400	205
ProximalPhalanxOutlineCorrect	Image	2	80	600	291
ProximalPhalanxTW	Image	6	80	400	205
RefrigerationDevices	Device	3	720	375	375
Rock	Spectrum	4	2844	20	50
ScreenType	Device	3	720	375	375
SemgHandGenderCh2	Spectrum	2	1500	300	600
SemgHandMovementCh2	Spectrum	6	1500	450	450
SemgHandSubjectCh2	Spectrum	5	1500	450	450
ShakeGestureWiimoteZ	Sensor	10	$\leq$19	50	50
ShapeletSim	Simulated	2	500	20	180
ShapesAll	Image	60	512	600	600
SmallKitchenAppliances	Device	3	720	375	375
SmoothSubspace	Simulated	3	15	150	150
SonyAIBORobotSurface1	Sensor	2	70	20	601
SonyAIBORobotSurface2	Sensor	2	65	27	953
StarLightCurves	Sensor	3	1024	1000	8236
Strawberry	Spectro	2	235	613	370
SwedishLeaf	Image	15	128	500	625
Symbols	Image	6	398	25	995
SyntheticControl	Simulated	6	60	300	300
ToeSegmentation1	Motion	2	277	40	228
ToeSegmentation2	Motion	2	343	36	130
Trace	Sensor	4	275	100	100
TwoLeadECG	ECG	2	82	23	1139
TwoPatterns	Simulated	4	128	1000	4000
UMD	Simulated	3	150	36	144
UWaveGestureLibraryAll	Motion	8	945	896	3582
UWaveGestureLibraryX	Motion	8	315	896	3582
UWaveGestureLibraryY	Motion	8	315	896	3582
UWaveGestureLibraryZ	Motion	8	315	896	3582
Wafer	Sensor	2	152	1000	6164
Wine	Spectro	2	234	57	54
WordSynonyms	Image	25	270	267	638
Worms	Motion	5	900	181	77
WormsTwoClass	Motion	2	900	181	77
Yoga	Image	2	426	300	3000
