# Supplementary figures and images for: An empirical survey of data augmentation for time series classification with neural networks
Source: PLoS One. 2021 Jul 15;16(7):e0254841. doi: 10.1371/journal.pone.0254841 (PMC8282049; doi:10.1371/journal.pone.0254841)

MLP

VGG

ResNet

LSTM

BLSTM

LSTM-FCN

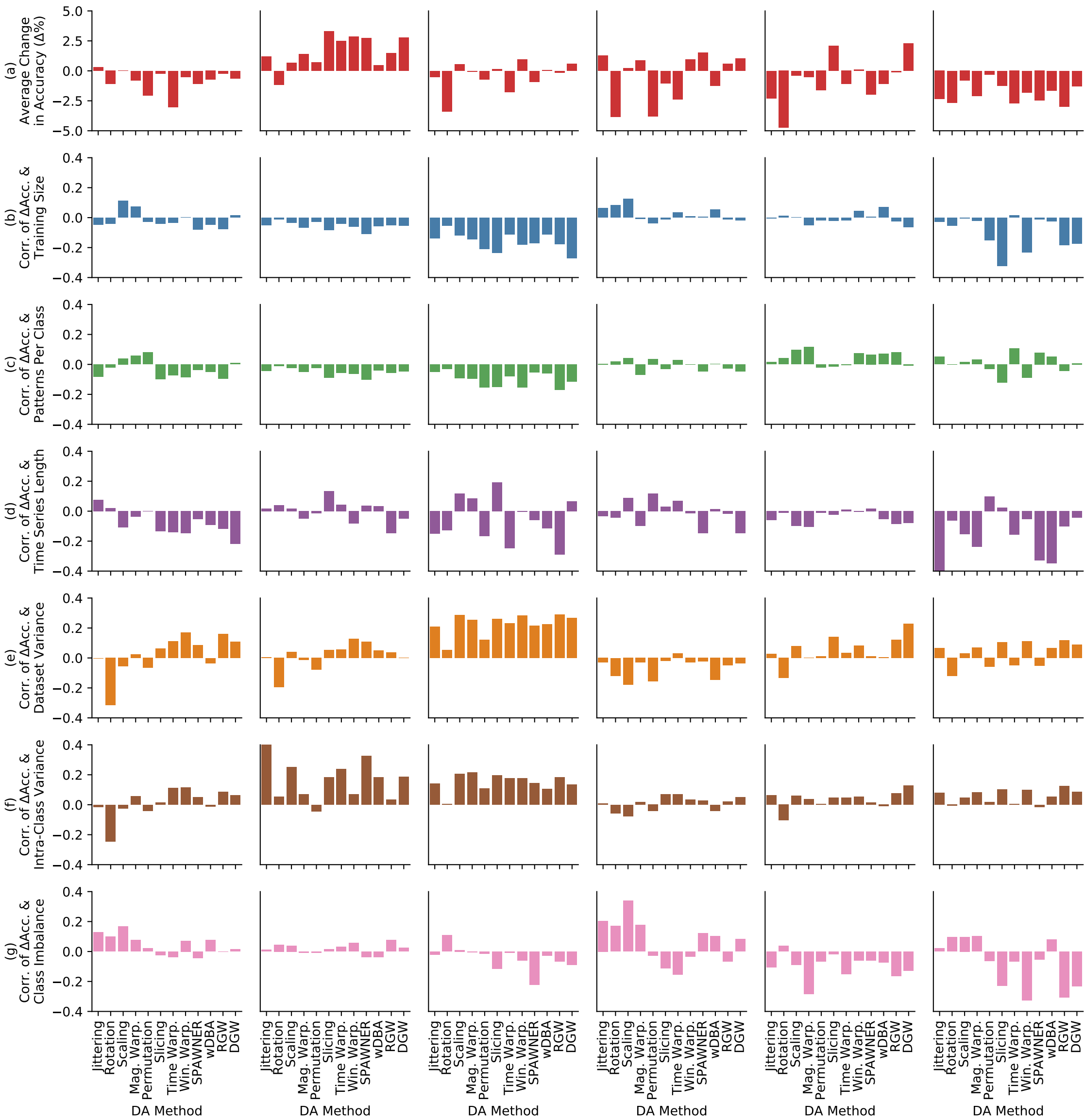

Supplement: S1 Fig — The top row in red is the change in accuracy and the subsequent rows are the correlations. (PDF) [file pone.0254841.s002.pdf]
